# Apolipoprotein E gene allele 4 and amyloid-beta mediate tau-related network breakdown

**DOI:** 10.1093/braincomms/fcaf404

**Published:** 2025-10-15

**Authors:** Fardin Nabizadeh

**Affiliations:** School of Medicine, Iran University of Medical Sciences, Tehran 14535, Iran

**Keywords:** Alzheimer’s disease, tau, fMRI, apolipoprotein E (APOE) ε4, amyloid-beta

## Abstract

There have been reports of altered functional connectivity in Alzheimer's disease, which is associated with the buildup of pathogenic proteins in the brain, including neurofibrillary tau tangles and amyloid-beta plaques. It is believed that the tau aggregates are the main driver of functional disconnection and resulted in cognitive decline in Alzheimer's disease. Tau propagates through connected neurons, a phenomenon often described as the ‘prion-like’ properties of tau, which can locally result in functional connectivity disruption. Apolipoprotein E gene allele 4 status and amyloid-beta are accelerating factors for tau-related pathological changes in Alzheimer's disease. However, the potential role of apolipoprotein E gene allele 4 and amyloid-beta in mediating the tau-related functional disconnection is not clear. I aimed to investigate the mediating effect of apolipoprotein E gene allele 4 and amyloid-beta on the local association of tau spreading on functional connections. I analysed follow-up resting-state functional MRI (fMRI) (non-baseline visit) and longitudinal tau-PET data from 211 subjects from the Alzheimer's Disease Neuroimaging Initiative (ADNI) database and 138 healthy elderly individuals from the Harvard Aging Brain Study (HABS). The follow-up resting-state fMRI (non-baseline visit) was studied in order to study the time needed effect of tau pathology. The top 10 regions with the highest probability-weighted SUVR values using Gaussian mixture models were selected as individual-level tau-PET epicentres. I looked at how the relationship between functional connectivity to epicentres and individualized connectivity-related tau spreading was mediated by amyloid-beta status and the apolipoprotein E gene allele 4 genotype. Higher rates of tau aggregation accumulation were seen in areas with stronger connectedness (shorter distance-based connectivity) to the baseline-defined tau epicentres. Moreover, the association between functional connectivity to epicentres and tau spreading through functional connections was mediated by apolipoprotein E gene allele 4 and amyloid-beta status in both dataset’s participants. Tau aggregates spread through functional connections and locally disrupt connectivity between tau epicentre and non-epicentre regions, which is mediated in apolipoprotein E gene allele 4 carriers and amyloid-beta-positive participants. These findings have implications for trial designs, proposing that apolipoprotein E gene allele 4 carriers and amyloid-beta-positive participants might need earlier intervention to attenuate tau spreading and tau relative functional disconnection.

## Teaser

Nabizadeh investigated the potential role of apolipoprotein E4 and amyloid-beta in mediating the tau-related functional disconnection using functional MRI data. It was found that tau aggregates spread through functional connections and locally disrupt connectivity between tau epicentre and non-epicentre regions, which is mediated in apolipoprotein E4 carriers and amyloid-beta positive participants.

## Graphical Abstract

**Graphical Abstract:**
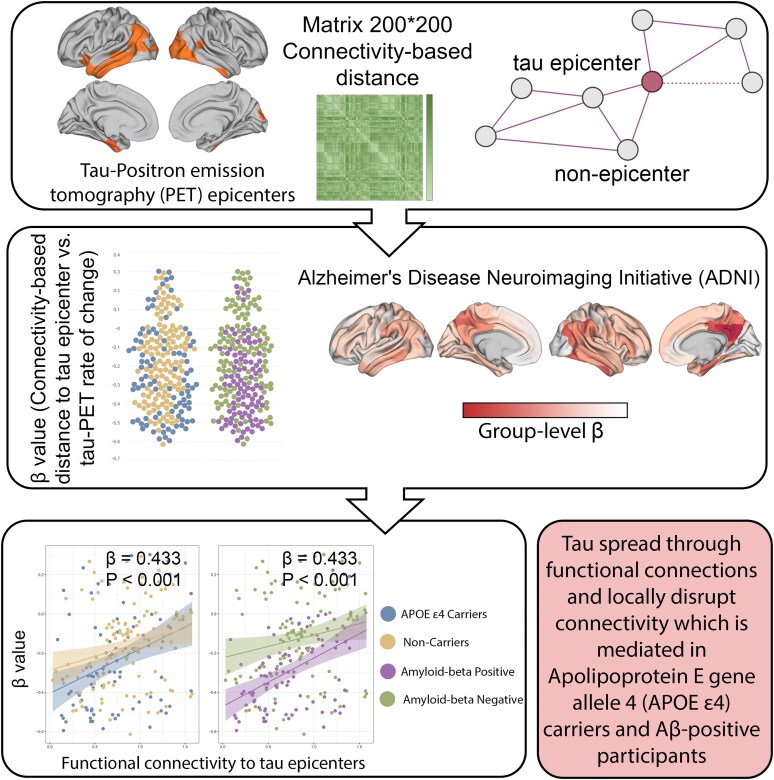

## Introduction

Alzheimer's disease is a common neurodegenerative disorder characterized by the gradual loss of neurons and synapses, along with disruption in neuronal connectivity. These aberrant characteristics disrupt the anatomical and functional integrity of brain networks, which has a significant impact on behaviour and cognition. Clinically, the most prevalent early symptom of early-stage Alzheimer's disease is memory impairment, which is associated with medial temporal lobe neurodegeneration.^1,2^ As the disease worsens, tau aggregates eventually affect the majority of the cortical regions, extending to the temporal and parietal cortices. Other cognitive domains, such as language, attention, executive skills and visuospatial abilities, also show up in Alzheimer's disease.^3^ The two neuropathological features that biologically define Alzheimer's disease are the abnormal accumulation of extracellular amyloid-β (Aβ) plaques and intracellular tau neurofibrillary tangles.^3^ Current research suggests that Aβ buildup begins in the medial frontal and medial parietal cortices of the default-mode network (DMN).^4^ In contrast, tau deposition begins in the medial temporal lobe memory system and progresses from the entorhinal cortex (EC) to the hippocampus and parahippocampal cortex before spreading to other areas of the brain.^5,6^ Experimental studies indicate that the anatomical pattern of tau propagation may be partially attributable to the release of misfolded tau from neurons, which is subsequently taken up by connected cells.^7^ This process facilitates the interneuronal spread of tau, potentially recruiting endogenous tau into a misfolded state—a phenomenon often described as the ‘prion-like’ properties of tau. Clinical studies combining fMRI and tau-PET imaging confirmed the tau spreading through the functional connections.^8-10^ A recent study demonstrated that the presence of cortical Aβ significantly increased the likelihood of tau spreading beyond the EC.^8^ A longitudinal study that combined tau–Aβ-PET with diffusion tensor imaging (DTI) of large fibre tracts provided additional evidence that tau pathology is propagated into the cortex in an Aβ-dependent manner, through direct neuronal connections rather than expansion into adjacent, unaffected regions. The study found that tau spreads into the posterior cingulate cortex (PCC) via the cingulum bundle, a major white matter tract that connects the hippocampus with the cingulate gyrus.^11^ The results of this study suggest that Aβ-associated tau accumulation is accelerated by the apolipoprotein E (APOE ε4) allele, the most significant risk factor of sporadic late-onset Alzheimer's disease, even at lower levels of Aβ, indicating that APOE ε4 may promote earlier Aβ-driven tau propagation across interconnected brain regions.

Both Aβ and pathological tau burdens have been shown to affect brain network connectivity.^12,13^ Alzheimer's disease pathogenesis is linked to changes in the DMN's functional connectivity (FC), which can occur years before cognitive symptoms appear.^14,15^ The research has documented a general decrease in within-network connectivity in the DMN among people who are Aβ-positive.^4,16^ However, changes in DMN connectivity appear to vary according to the level of Aβ burden and may not be uniform across different regions within the DMN. Tau is more strongly associated with cognitive decline than Aβ, and it is generally believed to be the primary cause of Alzheimer's disease neurodegeneration.^17,18^ Increased tau-PET levels have been linked to decreased connection between highly interconnected hubs and within different brain networks, according to a study by Cope *et al*.^19^ Additionally, a study conducted on cognitively normal older individuals found a positive correlation between elevated global amyloid levels and localized tau levels and enhanced posterior DMN connections to non-DMN regions.^20^ Although it can be drawn that tau accumulation is associated with reduced FC, we lack a fundamental understanding of the details of this relationship.^19,21,22^ One possible explanation is that the tau aggregates spread through neuronal connections and locally disrupt synaptic dysfunction, leading to decreased FC. In my previous study, I demonstrated that FC disruption in the pathway of tau spreading is locally associated with tau spreading through that connection.^23^ The crux of this hypothesis rests on the assumption that the neuronal networks act as ‘passive’ anatomical conduits for tau transportation.

A previous study demonstrated that the key brain network changes are more closely linked to tau pathology, not Aβ, among APOE ε4 carriers.^24^ In addition, another study found that APOE ε4 exacerbates tau-mediated neurodegeneration in mouse models.^25^ The presence of APOE ε4 may make neurons more susceptible to tau-related neurodegeneration, and the absence of APOE ε4 seems to have protective effects against neuronal loss. Similarly, the presence of Aβ is found to be permissive for tau-related hippocampal dysfunction and memory performance based on the results of a recent investigation.^26^ Furthermore, despite the similar level of tau pathology, non-demented individuals with pathological levels of Aβ showed higher cognitive impairment, which raises the possibility that the presence of Aβ may increase the toxicity of tau aggregates and downstream neurodegeneration. Taken together, there is converging evidence that links APOE ε4 and Aβ to exacerbated tau-related neurodegeneration and facilitated tau spreading. Yet, current human and animal investigations overwhelmingly stem from cross-sectional data, and a biologically plausible cascade model that investigates the pathological effect of tau aggregates spreading on FC of networks acted as a conduit and the potential role of APOE ε4 and Aβ in mediating the tau-related functional disconnection is still missing. To address this gap, I aimed to incorporate longitudinal molecular imaging and resting-state fMRI connectomics in two independent large-scale cohorts of elderly participants. First, I investigated whether the presence of APOE ε4 and Aβ can mediate the connectivity-based accumulation of tau aggregates. Second, I tested whether the spreading of tau pathology is locally associated with decreased FC. Lastly, I investigated the potential mediation role of APOE ε4 and Aβ on the tau spreading-related functional disconnection.

## Materials and methods

### Participants

#### Alzheimer's Disease Neuroimaging Initiative

Initiated in 2003 as a public–private collaboration, the Alzheimer's Disease Neuroimaging Initiative (ADNI) is a multicentre study. The main goal of ADNI has been to determine if it is possible to accurately monitor the development of moderate cognitive impairment (MCI) and early Alzheimer's disease by combining serial MRI, PET imaging, several biological markers and clinical and neuropsychological evaluations. Check out adni.loni.usc.edu for the latest information. The data used in this study came from ADNI3 (NCT02854033), which were approved by all applicable ADNI ethics committees. A total of 211 people from the ADNI database were included in the study. The availability of baseline 18F-Florbetapir or 18F-Florbetaben amyloid-PET, longitudinal 18F-Flortaucipir tau-PET imaging and follow-up resting-state fMRI data were all necessary to meet the inclusion criteria. It was necessary to gather all baseline images within a 12-month period. Whole-cerebellum normalized global amyloid-PET standardized uptake value ratios (SUVR) were used to assess Aβ status in accordance with predetermined guidelines and cutoff points (global AV45 SUVR > 1.11; global FBB SUVR > 1.08). Global amyloid-PET SUVRs were converted to the Centiloid scale to make cross-tracer comparability easier. Participants were classified as cognitively normal (CN; MMSE > 24, CDR = 0, non-depressed), mildly cognitively impaired (MCI; MMSE > 24, CDR = 0.5, objective memory impairment on the education-adjusted Wechsler Memory Scale II, preserved activities of daily living) or demented (MMSE 20–26, CDR > 0.5, meeting NINCDS/ADRDA criteria for probable Alzheimer's disease) based on their clinical status, which was evaluated in accordance with ADNI protocols. All individuals gave written informed permission, and the ADNI investigators at each participating location acquired ethical approval. To rule out any confounding effects related to the potentially protective ε2 allele, those with the APOE ε2/ε4 genotype were not included.^27^ Written consent to participate was given for each subject, and the study was performed according to the Helsinki ethical principles.

#### Harvard Aging Brain research

The Harvard Aging Brain research (HABS; https://habs.mgh.harvard.edu), a single-centre observational study carried out at Massachusetts General Hospital, provided me with 138 healthy volunteers. To better understand the preclinical stages of Alzheimer's disease, HABS is a longitudinal study that focuses on older people who were CN at the time of enrollment.^28^ To qualify as CN during screening, participants were required to exhibit normal performance on the MMSE, logical memory and CDR = 0, adjusted for age and education. Additionally, participants had to demonstrate the absence of significant depressive symptoms, as indicated by a geriatric depression scale score of <10/30. Participants who had baseline Pittsburgh compound B (PiB) amyloid-PET, follow-up resting-state fMRI, and available longitudinal tau-PET data were included. Additionally, information on APOE ε4 status (the presence or absence of the ε4 allele) was gathered. Written consent to participate was given for each subject, and the study was performed according to the Helsinki ethical principles.

### Image acquisition and processing

The provided material provides a complete description of the PET and MRI imaging acquisition methodologies used in the ADNI cohort (http://adni.loni.usc.edu/methods/documents/). Realignment, averaging, reslicing to a 1.5 mm³ voxel size and smoothing to an 8 mm³ full-width at half maximum resolution were among the image processing procedures that were carried out internally. The PET images and the corresponding structural T1-weighted MRI scans were spatially matched. SUVR PET images were then produced, employing the entire cerebellum for the florbetapir and florbetaben tracers and the inferior cerebellum as the reference region for the flortaucipir tracer. Additionally, each participant's T1-weighted MRI image was registered to the standardized Montreal Neurological Institute (MNI) template space using ANTs software version 2.3.1. To make further studies easier, the SUVR PET images were converted into the MNI space using the same registration parameters. Both structural MRI and functional PET data were spatially normalized thanks to this processing pipeline, enabling group-level comparisons and region-of-interest (ROI) studies within a common anatomical framework.

After the PiB-PET radiotracer injection, imaging was done with a 60-min dynamic capture to provide a composite measure of neocortical Aβ in the HABS. The ECAT EXACT HR+ scanner (Siemens) was used for imaging in accordance with a previously defined protocol.^29^ The acquired images were co-registered to the participants’ T1-weighted anatomical images using ROI based on Freesurfer (version 6.0). The obtained pictures were then mapped into native PET space using SPM12. Using the cerebellar grey matter as the reference region, the distribution volume ratio (DVR) of the composite neocortical Aβ measure was computed across the frontal, lateral temporal, parietal and retrosplenial (FLR) regions. Participants were subsequently classified into two groups, Aβ-positive and Aβ-negative, based on their baseline PiB-PET DVR measurements. A cutoff value of 1.2, determined from a Gaussian mixture model (GMM), was utilized to make this distinction.^29^ In the HABS, tau pathology was assessed using images acquired during either the 80–100 min or 75–105 min post-injection period following the administration of the FTP-PET tracer. These imaging procedures were conducted using the ECAT HR+ scanner (Siemens) in accordance with a previously established protocol.^30^

### tau-PET rate of changes and epicentres

In the current investigation, the tau-PET pictures were divided into 200 regions that corresponded to the Schaefer functional atlas, a well-known framework accessible via the Nilearn module (nilearn.datasets.fetch_atlas_schaefer_2018).^31^ After applying a grey matter mask to each ROI, the mean SUVR values were computed for each of these 200 regions, which encompass the whole neocortex. The purpose of this masking procedure was to reduce the impact of the white matter or CSF signal on the final SUVR results that were utilized in the analysis.

Each brain region was subjected to linear mixed-effects models with random slopes and intercepts in order to calculate the rate of change in the tau-PET signal over time. Time (measured in years from the baseline scan) was the independent variable in these models, and tau-PET SUVR was the dependent variable. Two to four tau-PET scans were performed on the participants. Each participant's annual rate of change in tau-PET SUVR, which indicates the pace at which tau aggregates accumulate over time, was represented by the slope that was obtained from these models.

Baseline tau-PET data were utilized to identify individual-level tau-PET epicentres by using GMM to identify regions with the highest risk of abnormality. Due to the skewed distribution of tau-PET, which indicates that many participants and brain regions do not display elevated tau aggregation, GMM was used to differentiate between specific and non-specific binding. After separating these signals using a two-component GMM on each area of the brain, the likelihood that a participant's tau-PET signal would fall into the ‘high’ tau distribution (right-most distribution) was determined.^10,23,32,33^ This probability avoids the requirement for preset thresholds by acting as a probabilistic indication of tau positivity. To properly capture the range of tau-PET SUVR values along the Alzheimer's disease continuum, the GMMs were fitted to the complete baseline tau-PET dataset (*n* = 349). Parcels in the somatomotor regions with low tau-PET SUVR, where the fit between one or two distributions was almost identical, were excluded from the epicentre selection process due to the difficulty of differentiating between high- and low-tau distributions. A refined SUVR score that lessened the influence of non-specific signals was then obtained by multiplying the tau-PET SUVR by the likelihood determined by the GMM. To find individual-level tau-PET epicentres, the top 10 sites with the highest probability-weighted SUVR values were selected for additional connectivity-based research.^10^

### Functional connectivity template and analyses

To investigate the potential relationship between tau-PET accumulation and functional brain architecture, a normative FC matrix was constructed using data from 69 CU individuals in the ADNI cohort who were Aβ negative and had low tau-PET binding (global SUVR < 1.3).^34,35^ Using follow-up fMRI data (non-baseline), a unique template matrix was also made for every research participant in order to examine FC between pairings of brain regions.^9,27^ The template matrix derivation process has already been explained. In short, functional images were realigned to the first volume, co-registered to native T1-weighted images and then additional preprocessing steps like detrending, band-pass filtering (0.01–0.08 Hz) and regressing out nuisance covariates (such as motion parameters, average white matter and CSF signals) were applied. Volumes with Power’s framewise displacement > 1 mm—calculated as the sum of the absolute frame-to-frame changes in the six rigid-body realignment parameters were excluded (‘scrubbed’) together with the two adjacent volumes. Participants with >30% censored volumes were excluded from further analysis.^36^ The Schaefer atlas was used to construct FC matrices with 200 parcels. To create subject-specific FC matrices, Fisher-z correlations between the time-series averaged across voxels within each ROI were calculated. After that, these discrete matrices were thresholded at 30% density and averaged. The average FC was converted into a distance-based connectivity matrix from the 69 CU individuals to produce a normative connection matrix, where better connectedness was indicated by shorter path lengths between ROIs.^37^

I determined the distance-based FC of brain areas to the previously identified tau epicentres in order to demonstrate a connection between normative FC and tau aggregation. Each region's connectivity-based distance to the tau epicentres across all non-epicentre brain regions (*n* = 190) was linked with the rate of tau-PET change in that region at both the group and individual levels. In order to capture connection-mediated tau spreading, this analysis assessed the correlation between tau-PET accumulation and connectivity to tau epicentres, reported as standardized beta (*β*)-values. Stronger connection (represented by smaller distance-based values) would be linked to increased tau-PET accumulation, as indicated by the expected negative *β*-values. Notably, identical results were obtained whether this procedure was replicated using a normative FC matrix based on partial correlations as a template or with the Euclidean distance between ROIs altered. Furthermore, to examine the relationship between the standardized *β*-value and FC to epicentres in each participant, only the FC between all non-epicentre sites (*n* = 190) and epicentres was included.

### Statistical analysis

*stats* v4.0.5, *lme4* v1.1-30 for linear mixed-effects models, *mediation* v4.5.0 for mediation analyses and *ggplot2* v3.3.6 for data visualization were among the essential packages used in all analyses, which were conducted using R version 4.0.5. Connectome WorkBench v1.5.0 was used to create brain representations.

I started by examining the relationship between the rate of tau aggregation buildup and the global Aβ load (Centiloid for ADNI and Neocortical Aβ DVR for HABS). To determine the relationship between connection to tau epicentres and the rate of tau-PET change, linear models were fitted across all non-epicentre regions for each participant. This produced a *β*-value for each person. Furthermore, using the *mediation* package in R, mediation analysis was used to examine the mediating influence of Aβ status and APOE ε4 allele on the relationship between tau-PET rate of change in non-epicentre regions and connectivity-based distance of these regions from tau epicentres. Age, sex, MMSE score and education were taken into account for each path in the mediation model, and 1000 bootstrap iterations were used to establish the significance of the mediation impact. To find the regional *β*-value, I performed a group-level study. For subject-specific epicentres, I looked into the relationship between the connectivity-based distance of the targeted region and the tau-PET rate of change in each region.

I investigated the association between each participant's *β*-value and FC to epicentres while adjusting for age, sex and education in order to test my main hypothesis. I first looked at the mediating role of Aβ status and APOE ε4 allele on the relationship between individual *β*-value and FC to epicentres adjusted for Aβ status and APOE ε4 allele in order to determine whether APOE ε4 carriers or Aβ-positive participants show accelerated functional disconnection as a result of tau spreading through functional connections. For every sample, a different 1000-iteration bootstrapped mediation model was used.

To generate brain maps that illustrate the influence of *β*-value (tau spreading) on functional disconnection, I first computed the residuals from linear regression models within each subject, where the independent variable was group-level *β*-value for each region, and the dependent variable was the FC to epicentres for each region. These residuals were studentized across brain regions in each subject. Given that the standardized residuals did not follow a normal distribution, the Wilcoxon rank-sum statistic was computed by combining the standardized residuals for each region across individuals. Using 1000 randomly permuted *β*-value maps for FC to the epicentre, the procedure was repeated to create a null distribution for the Wilcoxon statistic. *z*-Scores for the Wilcoxon statistic in relation to its null distribution were computed in order to evaluate the significance of the Wilcoxon maps. The Aβ and APOE ε4 groups underwent separate analysis.

Number of data underlying were derived from the same ADNI dataset as used in my previous publication,^23^ and the analytical approach was similar.

## Results

### Sample characteristics

**Figure 1:**
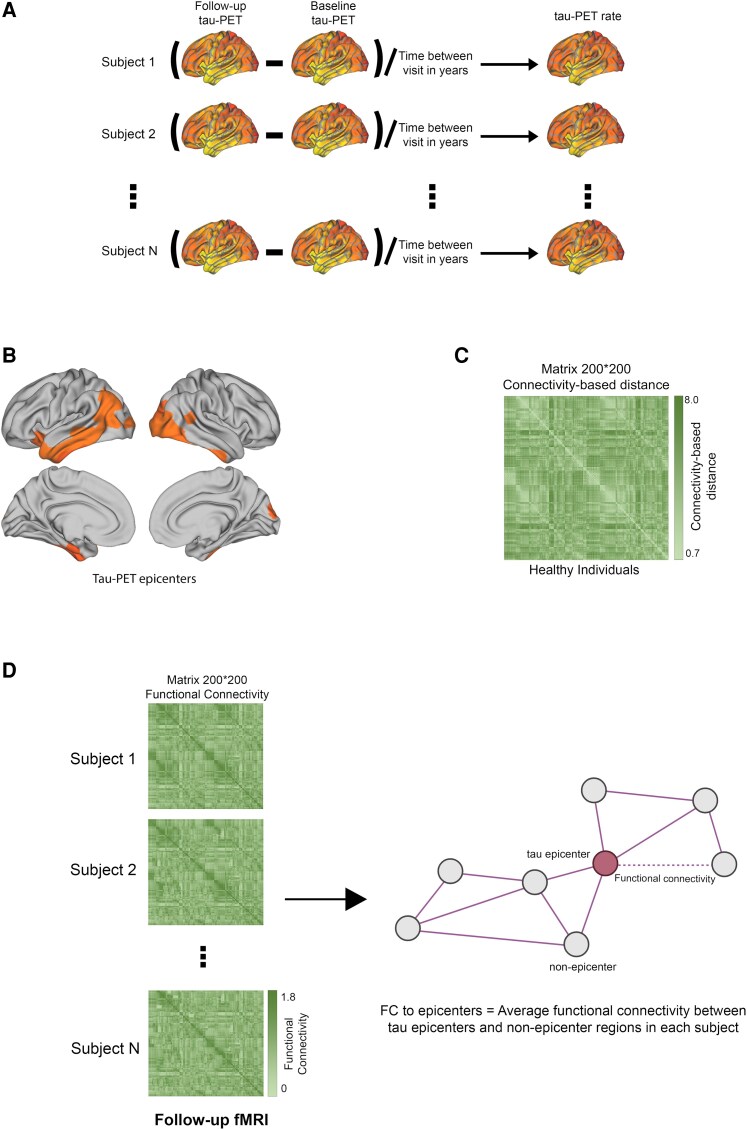
**Assessment of tau rate of change and FC template.** (**A**) The tau rate of change in each ROI was assessed by the difference between follow-up and baseline tau-PET, divided by the time difference between the two assessments. (**B**) The group-level tau-PET epicentres (top 5% of regions with highest tau-PET SUVR using Gaussian mixture model) are visualized on the glass brain. (**C**) Inter-regional FC distances were derived using data from 69 CN subjects from the ADNI dataset. Subject-specific connectivity matrices, which were constructed by applying Fisher-z transformations to the correlations of ROI-specific preprocessed fMRI time series, were averaged across individuals. These matrices were then thresholded at a 30% density, retaining only the top 30% of the strongest connections. Subsequently, these matrices were converted to connectivity-based distances, where a shorter distance indicates higher connectivity. (**D**) A 200 × 200 matrix representing inter-regional FC was constructed for each participant using follow-up fMRI data, excluding imaging data from baseline visits. For each subject, the average FC between non-epicentre regions and tau-PET epicentre regions was calculated, referred to as FC to epicentres. This measure directly reflects the FC of inter-regional pathways that potentially facilitate tau spreading from epicentre to non-epicentre regions, as posited by the hypothetical tau spreading model. fMRI, functional magnetic resonance imaging; PET, positron emission tomography; FC, functional connectivity.

In this study, I analysed data from 211 subjects from the ADNI database, each of whom had available follow-up resting-state fMRI (non-baseline visit) and longitudinal tau-PET data. The cohort comprised 20 CN and 74 MCI Aβ-negative individuals serving as a healthy group, 43 CN and 74 MCI Aβ-positive individuals representing the pre-dementia Alzheimer's disease spectrum (refer to Table 1 for baseline demographics). The mean tau-PET follow-up duration was 1.83 ± 1.23 and the average time interval between baseline tau-PET and follow-up visits of cross-sectional fMRI data was 1.94 ± 1.66 years. Also, the data of 138 healthy elderly individuals from HABS, including 42 CN Aβ-positive individuals, were entered into the current study. For the participants in the HABS, the average period between baseline tau-PET and follow-up visits of cross-sectional fMRI data was 2.53 ± 1.48 years, and the average tau-PET follow-up length was 4.04 ± 2.11. They used the Schaefer brain atlas to divide the PET data into 200 cortical ROIs.^31^ Tau accumulation within each brain region was quantified as the annualized rate of change in tau-PET signal (SUVR/year) (Fig. 1A).

**Table 1:** Demographic and clinical characteristics

Variable	ADNI (*n* = 211)	HABS (*n* = 138)
Age (years)	68.8 ± 6.2	71.2 ± 6.3
Female (%)	120 (57%)	78 (60%)
Education (years)	16.3 ± 2.5	16.8 ± 2.7
APOEε4 carriers (%)	85 (40%)	35 (25%)
Aβ-positive (%)	117 (55%)	42 (30%)
PACC-5	0.07 (0.71)	0.26 (0.64)
tau-PET follow-up time (years)	1.83 ± 1.23	4.04 ± 2.11
MCI (%)	74 (35%)	0

Data are presented as mean ± standard deviation unless specified otherwise. Abbreviations: APOEε4, apolipoprotein E genotype (carrying at least one ε4 allele); PACC, preclinical Alzheimer cognitive composite; PET, positron emission tomography; MCI, mild cognitive impairment.

### Functional connectivity template

The functional architecture of the brain plays an important role in regional tau buildup. I investigated whether the brain's FC is impacted by the connection between connectivity-based tau aggregate buildup. Using Gaussian mixture modelling at baseline, I first selected participant-specific tau-PET epicentres, or the top 10 locations with the highest tau-PET SUVR probability (Fig. 1B). In order to mimic the buildup of tau aggregates, I also developed a template of a 200 × 200 inter-regional matrix of the connectivity-based distance of healthy persons (Fig. 1C). The Schaefer brain atlas, which consists of 200 ROIs, was then used to preprocess follow-up resting-state fMRI data in order to produce subject-specific FC metrics (Fig. 1D).^31^ To concentrate solely on the connections implicated in tau spreading (Fig. 1D), FC to subject-specific tau epicentres were computed (Fig. 1B) using the 200 × 200 inter-regional FC matrix for each participant.

### Connectivity-based tau aggregates accumulation

**Figure 2:**
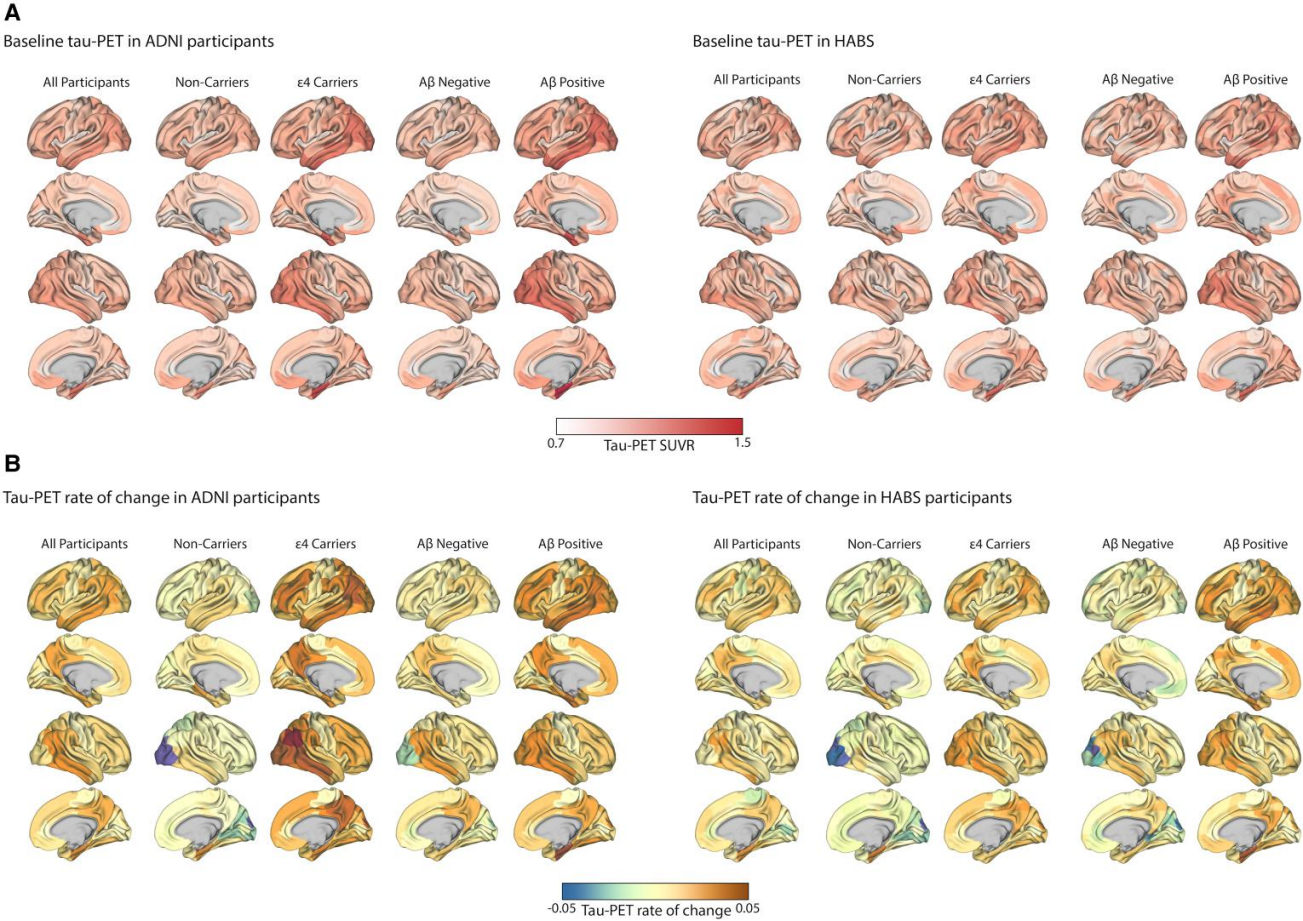
**Average spatial distribution of baseline and longitudinal tau-PET SUVRs stratified by APOE4 status and Aβ.** (**A**) Surface renderings of the average baseline tau-PET SUVR were generated for four groups: Aβ-negative (ADNI *n* = 94, HABS *n* = 96), Aβ-positive non-demented participants (ADNI *n* = 117, HABS *n* = 42), APOE ε4 carriers (ADNI *n* = 85, HABS *n* = 35) and non-carriers (ADNI *n* = 126, HABS *n* = 103). These renderings were mapped across the 200 parcels defined by the Schaefer 200-ROI atlas. (**B**) Surface rendering of tau rate of change derived by the difference between follow-up and baseline tau-PET, divided by the time difference between the two assessments for the same groups in (**A**). APOE, apolipoprotein E ε4; Aβ, amyloid beta; ROI, region of interest; PET, positron emission tomography.

**Figure 3:**
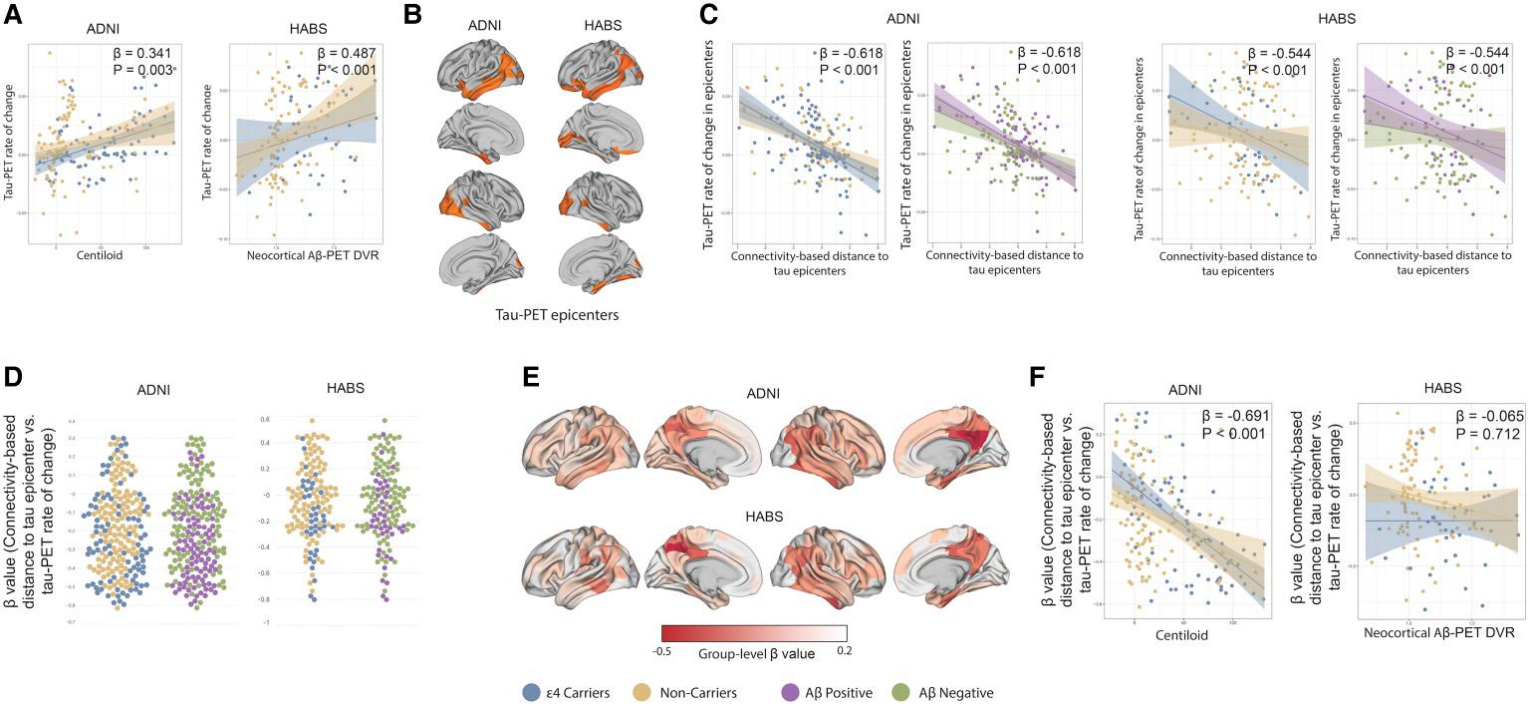
**Connectivity-based accumulation of tau aggregates.** (**A**) A scatter plot illustrates the associations between the tau-PET rate of change and the Aβ (Centiloid). Each dot represents a participant. The observed positive association indicates that higher Aβ levels are linked with the faster tau-PET accumulation (ADNI *n* = 204, HABS *n* = 137). (**B**) The group-level tau-PET epicentres (top 5% of regions with highest tau-PET SUVR using Gaussian mixture model) are visualized on the glass brain. (**C**) Interaction between connectivity-based distance with APOE ε4 and Aβ on the tau-PET rate of change in non-epicentre regions (ADNI *n* = 211, HABS *n* = 138). The analysis demonstrates the relationship between connectivity-based distance to individualized tau epicentres and the tau-PET rate of change across non-epicentre regions. The findings indicate that regions with closer FC distance to the epicentres exhibit a higher rate of tau-PET accumulation. Furthermore, there were significant between connectivity-based distance-by-ApoE4 status and connectivity-based distance-by-Aβ status interactions on tau-PET rate of change in non-epicentre regions. Data and analytical approach similar to my previous study for ADNI dataset (23). (**D**) Repeating the same analysis at the individual level (ADNI *n* = 211, HABS *n* = 138). The *β*-value obtained from the correlation analysis between the tau-PET rate of change in non-epicentre regions and the connectivity-based distance to epicentres for each individual is presented in a box plot (mean: −0.208). These results suggest that tau spreads through functional connections. A more negative *β*-value indicates a higher rate of tau accumulation in regions that are functionally closer to the tau epicentres. Data and analytical approach similar to my previous study for ADNI dataset (23). (**E**) Surface rendering of group-level *β*-value across all participants (ADNI *n* = 211, HABS *n* = 138). (**F**) A scatter plot of the association between Aβ levels (Centiloid) and the *β*-values (connectivity-based distance to tau epicentres versus tau-PET rate of change in non-epicentre) (ADNI *n* = 204, HABS *n* = 137). Each dot on the plot represents an individual. The observed negative association suggests that elevated Aβ levels are associated with a faster rate of tau accumulation in regions that are more functionally closer to the epicentres (tau spreading). All linear regressions performed were two-sided, without adjustment for multiple comparisons, and error bands correspond to the 95% confidence interval. The linear models adjusted for the effect of age, sex, MMSE score and education. APOE, apolipoprotein E ε4; Aβ, amyloid beta; ROI, region of interest; PET, positron emission tomography; FC, functional connectivity.

Figure 2 represents the baseline and rates of tau-PET aggregates accumulation, stratified by Aβ and APOE ε4 status. The distribution of baseline tau aggregates, as evaluated through tau-PET, mirrored the typical Alzheimer's disease deposition patterns (Fig. 2A). These were observed in the medial and lateral temporal lobes among the APOE ε4 non-carriers and Aβ-negative participants, extending to the lateral and medial parietal and lateral occipital regions during symptomatic Alzheimer's disease stages for APOE ε4 carriers and Aβ-positive participants from both ADNI and HABS. The longitudinal tau aggregate accumulation rate was most pronounced in the temporal lobe regions and extensive involvement, particularly in the medial parietal and lateral frontal regions (Fig. 2B). I investigated the relationship between global Aβ load (Centiloid for ADNI and Neocortical DVR for HABS) and the rate of tau aggregate accumulation. The linear regression model revealed a positive association between global Aβ and the faster accumulation of tau aggregates over time in both ADNI and HABS participants (Fig. 3A).

As has already been mentioned, tau accumulation can be greatly influenced by functional architecture. First, the Gaussian-mixture model is used to define tau-PET epicentres specific to each participant (Fig. 3B). Using the functional connectome of healthy individuals, I assessed the correlation between the strength of FC to these epicentres (connectivity-based distance) and the accumulation of tau aggregates in the remaining 190 locations. Regions with shorter distance-based connection, which indicates better connectedness to the epicentres, showed higher rates of formation of insoluble tau aggregates at the group level (Fig. 3C).

Next, the model was applied at the individual level using tau-PET epicentres particular to each participant. Stronger connection (shorter distance-based connectivity) to the baseline-defined tau epicentres was associated with higher rates of tau aggregation development (Fig. 3D). The correlation between tau aggregation buildup in non-epicentre locations and connectivity to the epicentres was confirmed by negative *β*-values (average: −0.208) (Fig. 3D). The group-level *β*-value cortical distribution was measured by conducting the same analysis for each region across all participants (Fig. 3E). Lastly, I investigated whether the spreading of tau through functional connections (indicated by negative *β*-values) is associated with global level Aβ pathology. I found that a higher Aβ level (Centiloid) is associated with a lower *β*-value (Connectivity-based distance to tau epicentres versus tau-PET rate of change in non-epicentre) in the ADNI dataset (Fig. 3F). However, the same analysis for the participants from the HABS dataset was not statistically significant.

### Aβ and APOE ε4 mediate the association between functional disconnection and connectivity-based tau spreading

**Figure 4:**
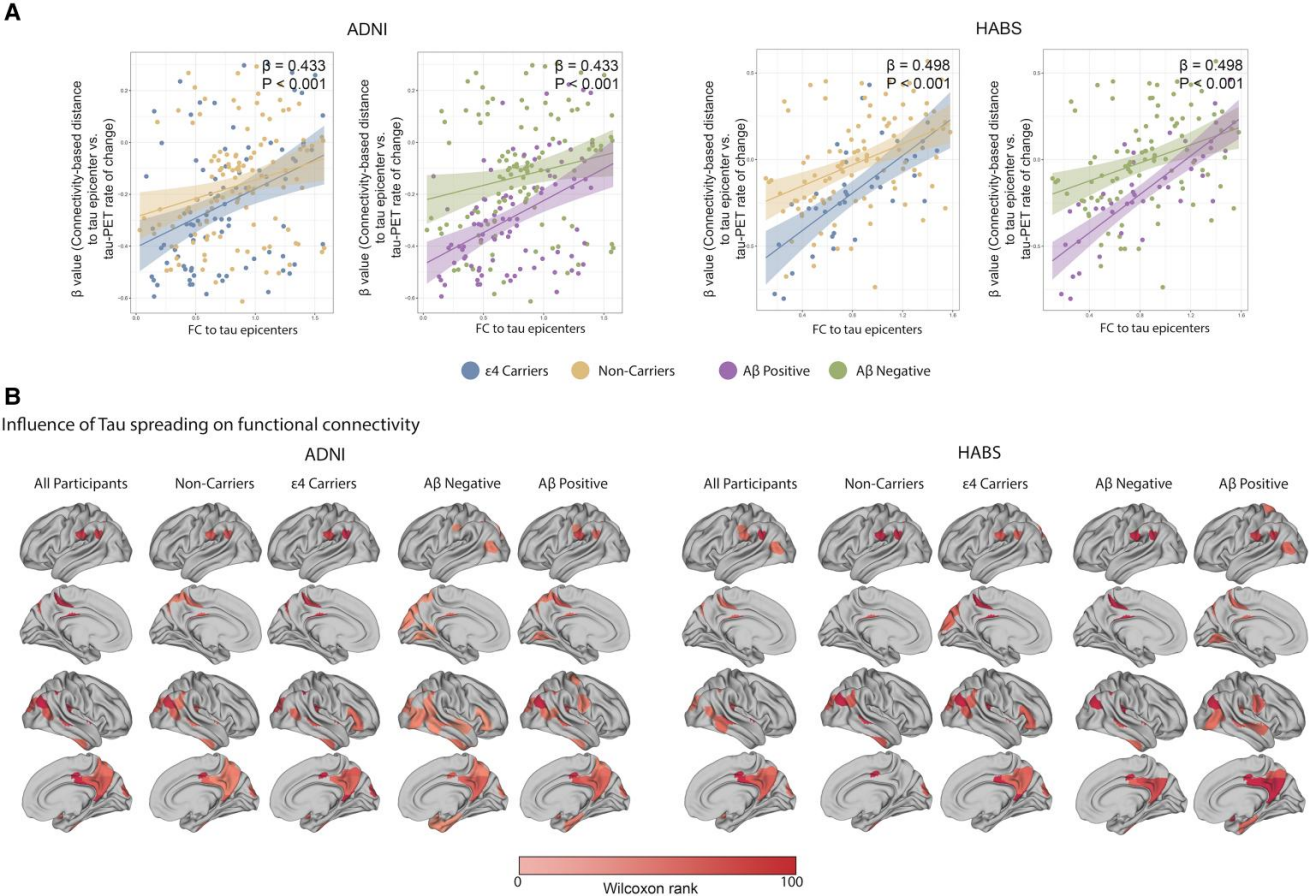
**Association between connectivity-mediated tau spreading and functional disconnection.** (**A**) The scatter plot illustrates the associations between the *β*-value (connectivity-based distance to tau epicentres versus tau-PET rate of change in non-epicentre) and FC to tau epicentres. Each dot represents a participant (ADNI *n* = 211, HABS *n* = 138). Interaction between *β*-value with APOE ε4 and Aβ on the FC to tau epicentres. The analysis demonstrates the association between connectivity-mediated tau spreading and weaker FC between tau epicentres and non-epicentre regions. Also, there were significant between *β*-value-by-ApoE4 status and *β*-value-by-Aβ status interactions on FC to tau epicentres. Data and analytical approach similar to my previous study for ADNI dataset (23). (**B**) Model-derived maps represent the influence of *β*-value (connectivity-mediated tau spreading) on FC to tau epicentres. The influence maps highlight brain regions where the *β*-value is significantly informative in explaining functional disconnection to the tau epicentre regions. These maps, rescaled to arbitrary units for visualization, depict the model residuals at each region due to the inclusion of *β*-value as predictors for FC to tau epicentres. *β*-Value influences are quantified using the Wilcoxon rank-sum statistics of the model’s residuals for a given region, with the maps displaying only regions with significant *z*-scores (*P* < 0.05) compared to null distributions: Aβ-negative (ADNI *n* = 94, HABS *n* = 96), Aβ-positive non-demented participants (ADNI *n* = 117, HABS *n* = 42), APOE ε4 carriers (ADNI *n* = 85, HABS *n* = 35) and non-carriers (ADNI *n* = 126, HABS *n* = 103). All linear regressions performed were two-sided, without adjustment for multiple comparisons, and error bands correspond to the 95% confidence interval. The linear models adjusted for the effect of age, sex, MMSE score and education. APOE, apolipoprotein E ε4; Aβ, amyloid beta; ROI, region of interest; PET, positron emission tomography; FC, functional connectivity.

I hypothesize that tau clumps moving via neuronal synapses might have a local effect on FC. I looked at the potential connection between FC to epicentres derived from follow-up fMRI data and connectivity-based tau aggregation accumulation (*β*-value) in order to demonstrate the local effects of tau spreading on the implicated connections. The results show that, for all groups examined in both datasets, a lower FC to epicentres is associated with a greater association between connectivity to the epicentre and the tau-PET rate of change in non-epicentre regions (*β*-value) (Fig. 4A).

Next, I hypothesized that the association between FC to epicentres and tau spreading might be mediated by Aβ and APOE ε4. Bootstrapped mediation analyses indicated that the relationship between FC to epicentres and tau spreading (*β*-value) was mediated by APOE ε4 status in ADNI participants (average causal mediation effect: *β* = 0.14; 95% CI, 0.06–0.27; *P* < 0.001) (Fig. 4A). Repeating the mediation analysis showed that the association between FC to epicentres and tau spreading is mediated by Aβ status ADNI participants (average causal mediation effect: *β* = 0.19; 95% CI, 0.08–0.32; *P* < 0.001) (Fig. 4A). The same analyses in the HABS participants confirms the previous results and showed mediation effect for APOE ε4 (average causal mediation effect: *β* = 0.17; 95% CI, 0.03–0.31; *P* < 0.001) and Aβ status (average causal mediation effect: *β* = 0.19; 95% CI, 0.02–0.33; *P* < 0.001) on the relationship between FC to epicentres and tau spreading (*β*-value) (Fig. 4A).

Lastly, by determining which brain regions had the best explanatory power, I evaluated the extent to which connectivity-based tau spreading (*β*-value) influenced FC to epicentres across different brain regions. I normalized residuals at each brain area, where each residual denoted an unexplained functional disconnection. I then used 1000 randomly shuffled *β*-value maps to fit the model and calculated the Wilcoxon rank-sum statistics of the population residuals for each region to produce a null distribution of Wilcoxon statistics. Brain areas exhibiting substantial residual improvements (*P* < 0.05) above the null distributions were filtered using this permutation test. These maps highlight regions where FC to epicentres are significantly better explained by connectivity-based tau spreading (*β*-value), rather than regions with the highest *β*-value. Figure 4B summarizes the connectivity-based tau spreading influence maps stratified by Aβ and APOE ε4 status.

## Discussion

**Figure 5:**
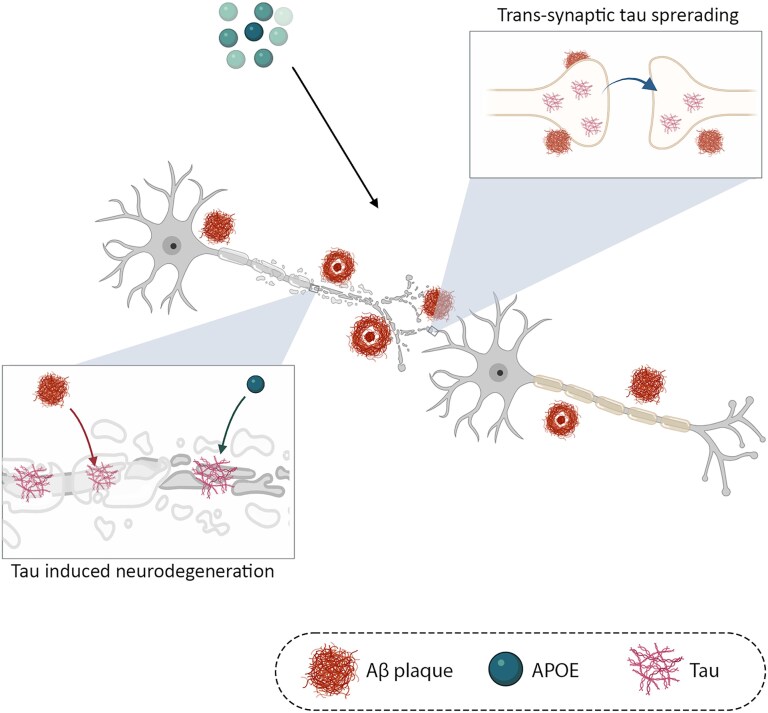
**Proposed model of functional disconnection in tau spreading pathways.** Tau aggregates are spread through connections, leading to local disruption in FC, which is accelerated in APOE ε4 carriers and those with pathological levels of Aβ. APOE, apolipoprotein E ε4; Aβ, amyloid beta; FC, functional connectivity.

Using the longitudinal molecular imaging and resting-state fMRI data of two large independent cohorts of elderly participants, I demonstrated an association between the tau aggregate rate of change and lower connectivity-based distance to the tau epicentres. It is in line with the previous studies found that the tau aggregates spread through neuronal connections and regions with higher connectivity to the other regions are more prone to be affected by tau pathology.^9,10,19,23^ Also, I found that the APOE ε4 and Aβ status mediates faster tau accumulation across functional connections. The major aim of the present study was to investigate the mediating effect of APOE ε4 and Aβ plaques on the association between tau pathology and downstream neurodegeneration indicated by functional connectivity. I showed that tau spreading and reduced FC changes in networks that acted as conduits for protein propagation, which was mediated by APOE ε4 and Aβ plaques in the ADNI and HABS cohort. This suggests the potential effect of both APOE ε4 and Aβ on the tau pathology first by accelerating the spreading of tau aggregates accumulation through neuronal connections and second, by exacerbating the effect of tau on the functional connections, possibly contributing to the spreading process (Fig. 5).

Recent studies provided consistent evidence to support the notion that APOE ε4 and Aβ accelerate tau accumulation.^38^ It is possible that the accelerated tau progression observed in APOE ε4 carriers may be partially attributable to higher Aβ accumulation.^39^ Also, there is evidence revealing that APOE ε4 was associated not only with faster tau accumulation driven by elevated Aβ levels but also showed synergistic interactions with Aβ in accelerating tau spreading.^40^ The synergic interaction between APOE ε4 and Aβ on tau accumulation is supported by my findings. Investigating the tau spreading through functional connections (*β*-value) showed that APOE ε4 has a mediating effect on the association between connectivity-based tau aggregate accumulation and Aβ deposition. Transneuronal tau spread may be affected by changes in remote connectivity by Aβ accumulation in areas connected to the EC, where tau pathology often begins.^41^ This process could be accelerated in APOE ε4 carriers, who, based on electrophysiological and fMRI data, show hyperconnectivity in regions with significant Aβ deposits compared to non-carriers.^42,43^ This Aβ-driven hyperconnectivity may enhance the spread of tau to other cortical regions in APOE ε4 carriers.

APOE ε4 and Aβ both mediate the relationship between tau spreading and reduced FC between tau epicentre and non-epicentre regions in the ADNI cohort, which is the main finding of the current investigation. Restoring neuronal energy balance might lessen the impact of early local buildup of pathogenic tau in axons, which causes presynaptic dysfunction, decreased neuronal activity, and severe behavioural deficits in mice.^44^ Additionally, the mislocalization of tau to dendritic spines has been shown to contribute to synaptic dysfunction, and there is evidence indicating that pathological tau diminishes overall network activity.^45^ Aβ buildup facilitates tau's expansion into the posterior cingulate cortex (PCC) through the cingulum bundle, a crucial white matter tract that connects the hippocampus and the cingulate gyrus, according to a study conducted on older persons.^8^ Episodic memory impairment was closely linked to the convergence of tau and Aβ in the PCC during a 6-year period.^11^ These results are consistent with a PET investigation in CN subjects that shown that tau and Aβ interaction, not just either disease, accelerated cognitive deterioration.^46^ Furthermore, in older persons who were CN, the presence of Aβ accelerated the accumulation of tau in the inferior temporal cortex, and after a 7-year follow-up, tau deposition was associated with cognitive deterioration.^47^ In a CSF biomarker study of people aged 50–90, it was further confirmed that tau and Aβ work together to predict memory decline. Total tau and phosphorylated tau (p-tau) levels only correlated with cognitive performance when Aβ deposition, as indicated by low CSF Aβ, was also present.^48^ The effect of tau and APP-Aβ co-expression on neuronal function has been investigated in two recent investigations. A study that examined the impact of tau and APP-Aβ co-expression on neuronal function showed that tau inhibits Aβ-induced neuronal hyperexcitability, which in turn lowers neuronal activity. These results suggest antagonistic effects of tau and Aβ on neuronal circuit activation.^49^ However, further analysis uncovered synergistic interactions at the circuit level, as tau-induced hyperexcitability was significantly accelerated in APP/PS1-rTg4510 mice compared to their rTg4510 counterparts. Combining my findings, Aβ and tau may interact and produce compounded effects of cell toxicity resulting in disruption in functional connections.

My analyses in which I investigated the mediation effect of APOE ε4 on the association between global Aβ and longitudinal tau, suggest a possible synergic effect between APOE ε4 and Aβ plaques. Furthermore, APOE ε4 mediated the tau-related disruption in FC. A study by Shi *et al*.^25^ demonstrated that APOE significantly influences neurodegeneration within the context of tau pathology, independently of Aβ. Specifically, APOE ε4 exacerbated neurodegeneration, while the absence of APOE ε4 has strong neuroprotective effects. This is likely a result of APOE's early modulation of tau pathology, leading to the emergence of distinct tau species with varying degrees of neurotoxicity. Also, APOE ε4 plays a critical role in P301S neuronal death. While APOE, particularly APOE ε4, seemed to increase neuronal sensitivity to degeneration in the presence of abnormal tau buildup, its absence appeared to have a protective effect against neuronal death.^25^ Because of its increased innate immune reactivity, APOE ε4 increased the neuroinflammation that degenerating neurons had already caused.^50^ Recent studies have demonstrated that the depletion of microglia effectively prevents the APOE ε4-induced atrophy in the same mouse models expressing human tau. This indicates the potential involvement of a distinct neuroinflammatory process driven by tau pathology that is independent of Aβ. Another study revealed that the altered neuronal signalling associated with tau pathology may be mediated by exaggerated neuroinflammation in APOE ε4 carriers.^24^ In my study, instead of investigating global connectivity or within-network FC, I explored the potential effect of tau spreading on the connections that are possibly involved in the process of tau spreading and acted as a conduit. This can provide an opportunity to specifically focus on the local effect of tau aggregate accumulation on FC and explore the other factors that may increase the neurotoxicity of tau pathology. However, the complex interplay between tau, APOE ε4 and Aβ necessities the need for future studies potentially further parsing these effects.

The study's main strength is the combination of longitudinal and cross-sectional neuroimaging markers from two different cohorts. When interpreting the results of the current study, there are a few things to consider. First, my focus was on FC, which, while generally aligned with DTI-measured structural connectivity, is not entirely consistent.^51^ This discrepancy may be partially attributed to the technical limitations of DTI, particularly in detecting crossing fibers and short-range cortico-cortical connections.^52^ Furthermore, rather than using direct connections, fMRI assesses the temporal relationships between regions that are connected via multi-synaptic pathways.^53^ Furthermore, I completely omitted from my model areas that had a low detectable tau burden, primarily subcortical areas. Furthermore, the ADNI dataset's CU and MCI patients have a comparatively low burden of tau disease, which lowers the signal-to-noise ratio for tau-PET imaging at the lower end of the spectrum. As a result, the analysis can record scan noise instead of the tau pathology signal in PET imaging. Furthermore, selecting the CU and MCI participants from ADNI and healthy individuals from the HABS dataset prevents us from attributing the findings to the late stages of Alzheimer's disease. Another limitation that should be considered is that I used a normative FC template, which did not consider the inter-individual variation in network organizations. Also, Aβ-positive subjects and APOE ε4 carriers may have a degree of disruption in FC, which may affect the tau spreading. The study was also planned with the assumption that the main cause of the development of tau disease is transneuronal tau spreading. It does, however, recognize the possible contribution of other elements, including limited distribution, regional susceptibility and other underlying mechanisms, to the emergence of tau pathology and the ensuing disruption of FC. Additionally, the onset and rate of symptom progression in Alzheimer's disease may be influenced by additional modifying factors, such as resilience and cognitive reserve. To address individual differences in susceptibility and resilience, all analyses were statistically adjusted for years of education, a widely recognized proxy for these factors in Alzheimer's disease.^54^ However, we cannot entirely rule out the possibility that variations in reserve and resilience may further influence tau pathology and neurodegeneration. An explanation for the lack of association between Aβ burden and connectivity-based tau spreading is that HABS represents an earlier disease stage with overall lower and narrower amyloid burden, thereby reducing statistical power to detect effects that are more apparent in the older and more heterogeneous ADNI sample. Moreover, I found a significant association between neocortical Aβ-PET and the rate of change in tau-PET signal rather than contemporaneous network dysfunction, indicating that amyloid’s influence on tau propagation may unfold over time rather than manifest immediately in connectivity metrics. Finally, methodological differences, including scanner platforms, preprocessing pipelines and the substantially smaller number of HABS participants, could be the reasons. Together, these factors suggest that the absence of a significant Aβconnectivity-based tau spreading relationship in HABS reflects cohort composition and design constraints rather than a true biological dissociation.

## Conclusion

My independently validated results demonstrated that APOE ε4 and Aβ are associated with accelerated disruption in FC due to tau spreading. Tau aggregates spread through functional connections and locally disrupt connectivity between tau epicentre and non-epicentre regions, which is mediated in APOE ε4 carriers and Aβ-positive participants. These findings have implications for trial designs, proposing that APOE ε4 carriers and Aβ-positive participants might need earlier intervention to attenuate tau spreading and tau relative functional disconnection. My findings could be a starting point for future studies investigating the potential factors that may be involved in the vulnerability or susceptibility to tau-related neurodegeneration and resulted cognitive decline.

## Data Availability

The datasets generated and/or analysed during the current study are available in the ADNI repository, https://adni.loni.usc.edu/ and HABS, https://habs.mgh.harvard.edu. The datasets used and/or analysed during the current study are available from the corresponding author upon reasonable request. The code used in the current study is available at (https://github.com/fardinnabizadeh/Tauconnectivity).

## References

[1] Squire LR, Stark CE, Clark RE. The medial temporal lobe. Annu Rev Neurosci. 2004;27:279–306. 15217334 10.1146/annurev.neuro.27.070203.144130

[2] Dubois B, Villain N, Frisoni GB, et al Clinical diagnosis of Alzheimer's disease: Recommendations of the International Working Group. Lancet Neurol. 2021;20(6):484–496. 33933186 10.1016/S1474-4422(21)00066-1 PMC8339877

[3] Yu M, Sporns O, Saykin AJ. The human connectome in Alzheimer disease—Relationship to biomarkers and genetics. Nat Rev Neurol. 2021;17(9):545–563. 34285392 10.1038/s41582-021-00529-1 PMC8403643

[4] Palmqvist S, Schöll M, Strandberg O, et al Earliest accumulation of β-amyloid occurs within the default-mode network and concurrently affects brain connectivity. Nat Commun. 2017;8(1):1214. 29089479 10.1038/s41467-017-01150-x PMC5663717

[5] Buckner RL, DiNicola LM. The brain’s default network: Updated anatomy, physiology and evolving insights. Nat Rev Neurosci. 2019;20(10):593–608. 31492945 10.1038/s41583-019-0212-7

[6] Braak H, Braak E. Neuropathological stageing of Alzheimer-related changes. Acta Neuropathol. 1991;82(4):239–259. 1759558 10.1007/BF00308809

[7] Jucker M, Walker LC. Propagation and spread of pathogenic protein assemblies in neurodegenerative diseases. Nat Neurosci. 2018;21(10):1341–1349. 30258241 10.1038/s41593-018-0238-6 PMC6375686

[8] Adams JN, Maass A, Harrison TM, Baker SL, Jagust WJ. Cortical tau deposition follows patterns of entorhinal functional connectivity in aging. Elife. 2019;8:e49132. 31475904 10.7554/eLife.49132 PMC6764824

[9] Pichet Binette A, Franzmeier N, Spotorno N, et al Amyloid-associated increases in soluble tau relate to tau aggregation rates and cognitive decline in early Alzheimer's disease. Nat Commun. 2022;13(1):6635. 36333294 10.1038/s41467-022-34129-4 PMC9636262

[10] Nabizadeh F . sTREM2 is associated with attenuated tau aggregate accumulation in the presence of amyloid-β pathology. Brain Commun. 2023;5(6):fcad286. 37942087 10.1093/braincomms/fcad286 PMC10629471

[11] Jacobs HIL, Hedden T, Schultz AP, et al Structural tract alterations predict downstream tau accumulation in amyloid-positive older individuals. Nat Neurosci. 2018;21(3):424–431. 29403032 10.1038/s41593-018-0070-z PMC5857215

[12] Hansson O, Grothe MJ, Strandberg TO, et al Tau pathology distribution in Alzheimer's disease corresponds differentially to cognition-relevant functional brain networks. Front Neurosci. 2017;11:167. 28408865 10.3389/fnins.2017.00167 PMC5374886

[13] Hoenig MC, Bischof GN, Seemiller J, et al Networks of tau distribution in Alzheimer's disease. Brain. 2018;141(2):568–581. 29315361 10.1093/brain/awx353

[14] Sperling RA, Laviolette PS, O'Keefe K, et al Amyloid deposition is associated with impaired default network function in older persons without dementia. Neuron. 2009;63(2):178–188. 19640477 10.1016/j.neuron.2009.07.003 PMC2738994

[15] Sorg C, Riedl V, Mühlau M, et al Selective changes of resting-state networks in individuals at risk for Alzheimer's disease. Proc Natl Acad Sci U S A. 2007;104(47):18760–18765. 18003904 10.1073/pnas.0708803104 PMC2141850

[16] Hedden T, Van Dijk KR, Becker JA, et al Disruption of functional connectivity in clinically normal older adults harboring amyloid burden. J Neurosci. 2009;29(40):12686–12694. 19812343 10.1523/JNEUROSCI.3189-09.2009 PMC2808119

[17] La Joie R, Visani AV, Baker SL, et al Prospective longitudinal atrophy in Alzheimer's disease correlates with the intensity and topography of baseline tau-PET. Sci Transl Med. 2020;12(524):eaau5732. 31894103 10.1126/scitranslmed.aau5732 PMC7035952

[18] Iaccarino L, Tammewar G, Ayakta N, et al Local and distant relationships between amyloid, tau and neurodegeneration in Alzheimer's disease. Neuroimage Clin. 2018;17:452–464. 29159058 10.1016/j.nicl.2017.09.016 PMC5684433

[19] Cope TE, Rittman T, Borchert RJ, et al Tau burden and the functional connectome in Alzheimer's disease and progressive supranuclear palsy. Brain. 2018;141(2):550–567. 29293892 10.1093/brain/awx347 PMC5837359

[20] Quevenco FC, van Bergen JM, Treyer V, et al Functional brain network connectivity patterns associated with normal cognition at old-age, local β-amyloid, tau, and APOE4. Front Aging Neurosci. 2020;12:46. 32210782 10.3389/fnagi.2020.00046 PMC7075450

[21] Malpas CB, Saling MM, Velakoulis D, Desmond P, O'Brien TJ. Differential functional connectivity correlates of cerebrospinal fluid biomarkers in dementia of the Alzheimer's type. Neurodegener Dis. 2016;16(3-4):147–151. 26610265 10.1159/000438924

[22] Sintini I, Graff-Radford J, Jones DT, et al Tau and amyloid relationships with resting-state functional connectivity in atypical Alzheimer's disease. Cereb Cortex. 2021;31(3):1693–1706. 33152765 10.1093/cercor/bhaa319 PMC7869088

[23] Nabizadeh F . Disruption in functional networks mediated tau spreading in Alzheimer’s disease. Brain Commun. 2024;6(4):fcae198. 38978728 10.1093/braincomms/fcae198 PMC11227975

[24] Butt OH, Meeker KL, Wisch JK, et al Network dysfunction in cognitively normal APOE ε4 carriers is related to subclinical tau. Alzheimers Dement. 2022;18(1):116–126. 34002449 10.1002/alz.12375 PMC8842835

[25] Shi Y, Yamada K, Liddelow SA, et al Apoe4 markedly exacerbates tau-mediated neurodegeneration in a mouse model of tauopathy. Nature. 2017;549(7673):523–527. 28959956 10.1038/nature24016 PMC5641217

[26] Düzel E, Ziegler G, Berron D, et al Amyloid pathology but not APOE ε4 status is permissive for tau-related hippocampal dysfunction. Brain. 2022;145(4):1473–1485. 35352105 10.1093/brain/awab405 PMC9128811

[27] Corder EH, Saunders AM, Risch NJ, et al Protective effect of apolipoprotein E type 2 allele for late onset Alzheimer disease. Nat Genet. 1994;7(2):180–184. 7920638 10.1038/ng0694-180

[28] Dagley A, LaPoint M, Huijbers W, et al Harvard aging brain study: Dataset and accessibility. Neuroimage. 2017;144:255–258. 25843019 10.1016/j.neuroimage.2015.03.069 PMC4592689

[29] Mormino EC, Betensky RA, Hedden T, et al Synergistic effect of β-amyloid and neurodegeneration on cognitive decline in clinically normal individuals. JAMA Neurol. 2014;71(11):1379–1385. 25222039 10.1001/jamaneurol.2014.2031 PMC4293023

[30] Johnson KA, Schultz A, Betensky RA, et al Tau positron emission tomographic imaging in aging and early Alzheimer disease. Ann Neurol. 2016;79(1):110–119. 26505746 10.1002/ana.24546 PMC4738026

[31] Schaefer A, Kong R, Gordon EM, et al Local-global parcellation of the human cerebral cortex from intrinsic functional connectivity MRI. Cereb Cortex. 2018;28(9):3095–3114. 28981612 10.1093/cercor/bhx179 PMC6095216

[32] Vogel JW, Iturria-Medina Y, Strandberg OT, et al Spread of pathological tau proteins through communicating neurons in human Alzheimer’s disease. Nat Commun. 2020;11(1):2612. 32457389 10.1038/s41467-020-15701-2 PMC7251068

[33] Franzmeier N, Dewenter A, Frontzkowski L, et al Patient-centered connectivity-based prediction of tau pathology spread in Alzheimer's disease. Sci Adv. 2020;6(48):eabd1327. 33246962 10.1126/sciadv.abd1327 PMC7695466

[34] Franzmeier N, Neitzel J, Rubinski A, et al Functional brain architecture is associated with the rate of tau accumulation in Alzheimer’s disease. Nat Commun. 2020;11(1):347. 31953405 10.1038/s41467-019-14159-1 PMC6969065

[35] Maass A, Landau S, Baker SL, et al Comparison of multiple tau-PET measures as biomarkers in aging and Alzheimer's disease. Neuroimage. 2017;157:448–463. 28587897 10.1016/j.neuroimage.2017.05.058 PMC5814575

[36] Power JD, Mitra A, Laumann TO, Snyder AZ, Schlaggar BL, Petersen SE. Methods to detect, characterize, and remove motion artifact in resting state fMRI. Neuroimage. 2014;84:320–341. 23994314 10.1016/j.neuroimage.2013.08.048 PMC3849338

[37] Rubinov M, Sporns O. Complex network measures of brain connectivity: Uses and interpretations. Neuroimage. 2010;52(3):1059–1069. 19819337 10.1016/j.neuroimage.2009.10.003

[38] Steward A, Biel D, Dewenter A, et al Apoe4 and connectivity-mediated spreading of tau pathology at lower amyloid levels. JAMA Neurol. 2023;80(12):1295–1306. 37930695 10.1001/jamaneurol.2023.4038 PMC10628846

[39] Salvadó G, Grothe MJ, Groot C, et al Differential associations of APOE-ε2 and APOE-ε4 alleles with PET-measured amyloid-β and tau deposition in older individuals without dementia. Eur J Nucl Med Mol Imaging. 2021;48(7):2212–2224. 33521872 10.1007/s00259-021-05192-8 PMC8175302

[40] Therriault J, Benedet AL, Pascoal TA, et al APOEε4 potentiates the relationship between amyloid-β and tau pathologies. Mol Psychiatry. 2021;26(10):5977–5988. 32161362 10.1038/s41380-020-0688-6 PMC8758492

[41] Lee WJ, Brown JA, Kim HR, et al Regional Aβ-tau interactions promote onset and acceleration of Alzheimer's disease tau spreading. Neuron. 2022;110(12):1932–1943.e5. 35443153 10.1016/j.neuron.2022.03.034 PMC9233123

[42] Koelewijn L, Lancaster TM, Linden D, et al Oscillatory hyperactivity and hyperconnectivity in young APOE-ɛ4 carriers and hypoconnectivity in Alzheimer's disease. Elife. 2019;8:e36011. 31038453 10.7554/eLife.36011 PMC6491037

[43] Pihlajamäki M, Sperling RA. Functional MRI assessment of task-induced deactivation of the default mode network in Alzheimer's disease and at-risk older individuals. Behav Neurol. 2009;21(1):77–91. 19847047 10.3233/BEN-2009-0231 PMC5450588

[44] Dennissen FJ, Anglada-Huguet M, Sydow A, Mandelkow E, Mandelkow EM. Adenosine A1 receptor antagonist rolofylline alleviates axonopathy caused by human tau ΔK280. Proc Natl Acad Sci U S A. 2016;113(41):11597–11602. 27671637 10.1073/pnas.1603119113 PMC5068267

[45] Hoover BR, Reed MN, Su J, et al Tau mislocalization to dendritic spines mediates synaptic dysfunction independently of neurodegeneration. Neuron. 2010;68(6):1067–1081. 21172610 10.1016/j.neuron.2010.11.030 PMC3026458

[46] Sperling RA, Mormino EC, Schultz AP, et al The impact of amyloid-beta and tau on prospective cognitive decline in older individuals. Ann Neurol. 2019;85(2):181–193. 30549303 10.1002/ana.25395 PMC6402593

[47] Hanseeuw BJ, Betensky RA, Jacobs HIL, et al Association of amyloid and tau with cognition in preclinical Alzheimer disease: A longitudinal study. JAMA Neurol. 2019;76(8):915–924. 31157827 10.1001/jamaneurol.2019.1424 PMC6547132

[48] Timmers M, Tesseur I, Bogert J, et al Relevance of the interplay between amyloid and tau for cognitive impairment in early Alzheimer's disease. Neurobiol Aging. 2019;79:131–141. 31055223 10.1016/j.neurobiolaging.2019.03.016

[49] Busche MA, Wegmann S, Dujardin S, et al Tau impairs neural circuits, dominating amyloid-β effects, in Alzheimer models in vivo. Nat Neurosci. 2019;22(1):57–64. 30559471 10.1038/s41593-018-0289-8 PMC6560629

[50] Yuan P, Condello C, Keene CD, et al TREM2 haplodeficiency in mice and humans impairs the microglia barrier function leading to decreased amyloid compaction and severe axonal dystrophy. Neuron. 2016;90(4):724–739. 27196974 10.1016/j.neuron.2016.05.003 PMC4898967

[51] Honey CJ, Sporns O, Cammoun L, et al Predicting human resting-state functional connectivity from structural connectivity. Proc Natl Acad Sci U S A. 2009;106(6):2035–2040. 19188601 10.1073/pnas.0811168106 PMC2634800

[52] Abhinav K, Yeh FC, Pathak S, et al Advanced diffusion MRI fiber tracking in neurosurgical and neurodegenerative disorders and neuroanatomical studies: A review. Biochim Biophys Acta. 2014;1842(11):2286–2297. 25127851 10.1016/j.bbadis.2014.08.002

[53] Grandjean J, Zerbi V, Balsters JH, Wenderoth N, Rudin M. Structural basis of large-scale functional connectivity in the mouse. J Neurosci. 2017;37(34):8092–8101. 28716961 10.1523/JNEUROSCI.0438-17.2017 PMC6596781

[54] Stern Y, Arenaza-Urquijo EM, Bartrés-Faz D, et al Whitepaper: Defining and investigating cognitive reserve, brain reserve, and brain maintenance. Alzheimers Dement. 2018;16:1305–1311. 10.1016/j.jalz.2018.07.219 PMC6417987 30222945

